# Labeled dataset for training despeckling filters for SAR imagery

**DOI:** 10.1016/j.dib.2024.110065

**Published:** 2024-01-15

**Authors:** Rubén Darío Vásquez-Salazar, Ahmed Alejandro Cardona-Mesa, Luis Gómez, Carlos M. Travieso-González, Andrés F. Garavito-González, Esteban Vásquez-Cano

**Affiliations:** a Faculty of Engineering, Politécnico Colombiano Jaime Isaza Cadavid, Medellín, 48th Av, 7-151, Colombia; b Faculty of Engineering, Institución Universitaria Digital de Antioquia, Medellín, 55th Av, 42-90, Colombia; c Electronic Engineering and Automatic Department, IUCES, Universidad de Las Palmas de Gran Canaria, Las Palmas de Gran Canaria, Spain; d Signals and Communications Department, IDeTIC, Universidad de Las Palmas de Gran Canaria, Spain

**Keywords:** Speckle, Synthetic Aperture Radar (SAR), Image denoising, Supervised learning, Labeled dataset

## Abstract

When training Artificial Intelligence and Deep Learning models, especially by using Supervised Learning techniques, a labeled dataset is required to have an input with data and its corresponding labeled output data. In the case of images, for classification, segmentation, or other processing tasks, a pair of images is required in the same sense, one image as an input (the noisy image) and the desired (the denoised image) one as an output. For SAR despeckling applications, the common approach is to have a set of optical images that then are corrupted with synthetic noise, since there is no ground truth available. The corrupted image is considered the input and the optical one is the noiseless one (ground truth). In this paper, we provide a dataset based on actual SAR images. The ground truth was obtained from SAR images of Sentinel 1 of the same region in different instants of time and then they were processed and merged into one single image that serves as the output of the dataset. Every SAR image (noisy and ground truth) was split into 1600 images of 512 × 512 pixels, so a total of 3200 images were obtained. The dataset was also split into 3000 for training and 200 for validation, all of them available in four labeled folders.

Specifications Table

**Table:** 

Subject	Earth-Surface Processes, Applied Machine Learning, Global and Planetary Change
Specific subject area	Applied remote sensing, Synthetic Aperture Radar (SAR) imagery
Data format	Raw images in Tag Image File Format (.tiff)
Type of data	Folders of Images
Data collection	The raw images were downloaded from Sentinel-1 Synthetic Aperture Radar, in Level 1 Detected High-Res Dual-Pol (GRD-HD), polarization VV. The same region of Toronto (Canada) was selected on 10 different dates, every 12 days from Aug. 24th, 2022 to Dec. 22nd, 2022. The images were downloaded from [1]. Every image is 16,732 (height) by 26,019 (width).
Data source location	Continent: America Region: Toronto – Ontario – Canada The coordinates used in [1] to look for the images were: 43.68147575783798, −79.42260565653709
Data accessibility	Repository name: Mendeley Data Data identification number: 10.17632/2xf5v5pwkr.1 Direct URL to data: https://data.mendeley.com/datasets/2xf5v5pwkr/1 Instructions for accessing these data: Go to the URL and navigate and download the root folder and its subfolder. Take into account the dataset description.
Related research article	

## 1 Value of the Data

• The dataset provides 512 × 512 pairs of labeled (noisy and ground truth) images which will be useful for training despeckling filters by using supervised learning. From an appropriate dataset with high-quality ground truth data, a high performance despeckling model can be obtained.
• The dataset was built by using actual SAR imagery, in this case from Sentinel-1 radar, which is not the typical approach used in the design of speckle filters. In general, synthetic speckle modeled by a Gamma distribution is added to optical images.
• The dataset contains 1500 images for training and 100 for validation of every label, which, from our experience, seems enough for a deep learning model. However, these data could be integrated with other data, even synthetic, to obtain other results and make comparisons, and so, enlarging the database.
• The region selected to build the dataset is a region with important characteristics that make images very heterogeneous because it contains water, shore, rural and urban areas with man-made structures like bridges, buildings, highways, and so on.
• This dataset can be used by any researcher or engineer who wants to train his/her filtering models and use the ground-truth subset to validate and measure his results.
• The methodology used in this dataset can be easily replicated with information from other regions or sensors and get a lot more images to train bigger and more complex models. This methodology consists of the following steps: Download actual SAR images of the same region,rescalization to values from 0 to 255, registration with respect to a selected reference, multitemporal fusion and clip into small parts of 512 × 512 pixels.

## 2 Data Description

**Fig. 1:**
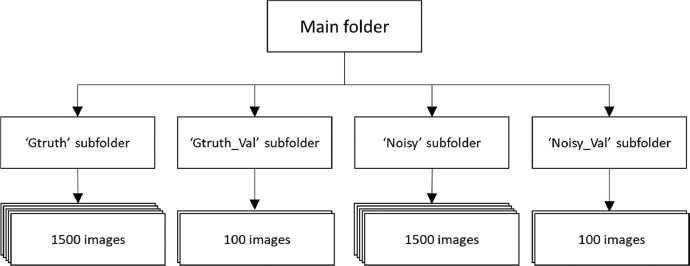
Structure of the dataset (main folder and subfolders).

The dataset is composed of four folders, namely: Ground Truth, Ground Truth validation, Noisy and Noisy Validation with 1500, 100, 1500 and 100 images respectively. Every image is 512 × 512 pixels, where every pixel is unique, which means that every pixel belongs only to one image because every image was extracted with no overlapping with the next one and its value corresponds to the intensity of the data signal back scattered. Every image is in Tag Image File Format (.tiff) and its weight is approximately 200 to 400 KB. The structure of the dataset is shown in Fig. 1.

The dataset does not have any annotations due it has the label text in the subfolder name, which is easy to read and implement in any programming language.

The sub-images in every folder come from a larger one, in this case, created with the SAR working in Sentinel-1. The original image downloaded is, 16,732 × 26,019 (height x width) pixels, which was clipped in 512 × 512 sub-images to create this dataset. The images were scaled with values from 0 (black) to 255 (white).

The designation of each image is *y_x.tiff*, where *y* and *x* are the *(x, y)* coordinates of the position of this sub-image in the original bigger one. For example, image *1024_15,360.png* was clipped starting from the coordinate *(x, y) = (15,360, 1024)* as far as the coordinate *(x* *+* *512, y* *+* *512) = (15,872, 1536)*. This name could be useful if the original image were rebuilt; if not, the names can be ignored.

## 3 Experimental Design, Materials and Methods

### 3.1 SAR imagery

Synthetic Aperture Radar (SAR) technology emits electromagnetic radiation at frequencies that vary from 300MHZ to 300 GHz, and receives back the signal after its interaction with objects on the earth's surface. This technology is relevant in the field of remote sensing because it can provide data in high resolution despite environmental conditions, which makes it crucial to analyze land cover, vegetation, water, and other remote sensing and environmental applications. SAR images are generated by a radar moving in a straight line while emitting an electromagnetic signal, which travels at the speed of light towards the surface. The signal then backscatters to the satellite, and its time delay is measured. So, the resulting images are built from the intensity and time calculated, which mainly depend on the roughness and electric properties of the surface under observation, by taking into consideration the speed of the satellite in its orbit [2].

These images, by their nature, include a noise called ‘speckle’, which is generated by the coherent illumination and the backscatter mechanisms. This speckle is also present in ultrasound images, and it is characterized by a granular pattern that generates strong interpretation challenges. From here, there are a lot more difficulties in the following stages: change detection, segmentation, and classification application over these images.

The process of removing or filtering the speckle has been an important field of study in recent years. The speckle is considered a multiplicative noise, it follows a Gamma distribution and is very hard to model. Different filters have been proposed in the literature, from the traditional ones [3], [4], [5], [6], [7], [8], to the ones based on artificial intelligence and deep learning, among others [9], [10], [11], [12], [13], [14], [15], [16].

### 3.2 Radar images from Sentinel-1

Sentinel-1 comprises a constellation of two polar-orbiting satellites, operating day and night and performing C-band synthetic aperture radar imaging, enabling them to acquire imagery regardless of the weather. Sentinel-1 will work in a pre-programmed operation mode to avoid conflicts and produce a consistent long-term data archive, built for applications based on long time series. It is designed to provide enhanced revisit frequency, coverage, timeliness, and reliability for operational services and applications requiring long time series. Sentinel-1A was launched on April 3rd, 2014, and Sentinel-1B on April 25th, 2016, both taken into orbit on a Soyuz rocket from Europe's Spaceport in French Guiana [17].

SAR images from Sentinel-1 can be acquired from different platforms; in this case, we used [1]. This platform allows looking for data with different criteria, including geographic search, dates, level of information, available polarizations, and so on. The technical specifications of the images used are shown in Table 1. The band used in the satellite instrument is C with a frequency of 5.4 GHz, the beam mode is Interferometric Wide Swath (IW), which is normally used for land observations, with a swath width of 250 km and a spatial resolution of 5 × 20 m. The polarization indicates the direction of the transmission and reception of the system, we used vertical linear transmission (V) and vertical linear reception (V).

**Table 1:** Technical specifications of downloaded images.

Item	Description
Mission	Sentinel 1A-1B
International Partner	ESA
Altitude/Inclination	693Km/98.2°
Band	C (5.4 GHz)
Beam Mode	Interferometric Wide Swath (IW)
Resolution	5 × 20 m
Revisit period	12 days (using together A and B, 6 days)
Processing Level	Level-1 Ground Range Detected (GRD)
Polarization	VV

### 3.3 Design of the dataset

#### 3.3.1 Rescale intensity

Images downloaded from ASF Data Search Vertex [1] of level L1 Detected High-Res Dual-Pol (GRD-HD) are in Tag Image File Format (.tiff), whose datatype is an unsigned integer of 16 bits (uint16) whose pixel values will be between 0 and 65,535. The overall image exhibits a diminished mean value, such that when displayed using any image viewer, the entire image appears predominantly black. Therefore, it is recommended to employ a rescaling technique to facilitate comprehensive visualization of the complete image. This is done as follows for every new pixel Eq. (1):

(1) $$I_{i,j}^{*}=\left\{ \begin{array}{c} 3*\bar{I},\;\\ I_{i,j},\\ 0, \end{array}\right.\begin{array}{c} \;if\;I_{i,j}>\bar{I}\;\\ \;if\;\text{min}\left(I\right)\leq I_{i,j}\leq \bar{I}\\ if\;I_{i,j}<min\left(I\right)\; \end{array}$$

**Fig. 2:**
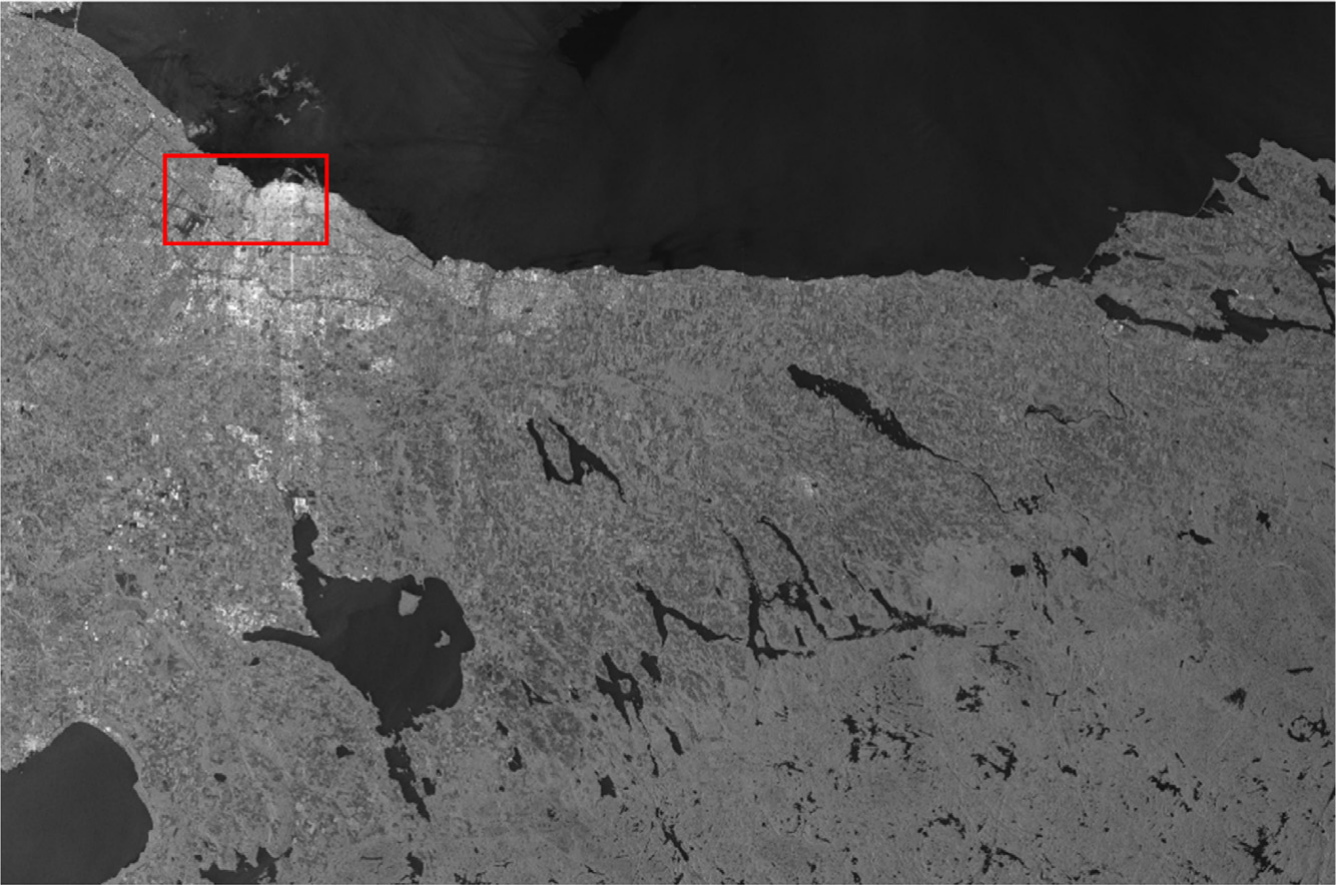
Example of actual SAR image rescaled taken from Sentinel-1 of the region of Toronto in September 2022.

**Fig. 3:**
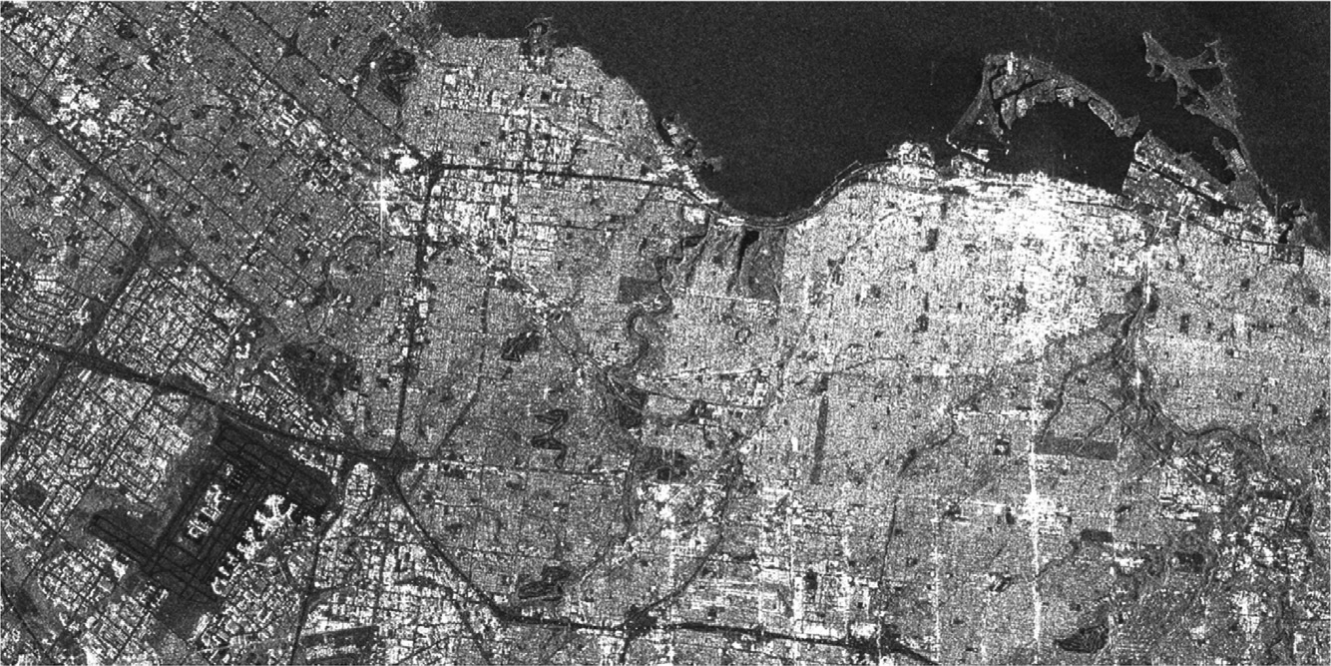
Zoom of the airport and coastal area in the SAR image rescaled taken from Sentinel-1 of the region of Toronto in September 2022.

Where $I_{i,j}^{*}$ is the pixel at coordinates (i,j) of the rescaled image and $\bar{I}$ is the mean of all pixels in the image I. After this rescalization process, the structure of the image is not modified, but all the values will be in the standard format *uint8*, with values from 0 to 255, which facilitates its visualization in any programming language, desktop software or application. This visualization is important when training despeckling models because it allows the expert to make a visual inspection of the results, prior to applying metrics for qualitative validation. An example of this result on a SAR image of the region of Toronto is shown in Figs. 2 and 3.

As shown in Fig. 3, the speckle is present in all the regions of the image, even in the water where the radar signal exhibits specular reflection (no return signal is at the SAR sensor, so nothing is detected, and consequently, the image pixels resemble full black and corrupted by the inherent speckle). Its presence will affect any post-processing task that is performed over the image.

#### 3.3.2 Image registration

The process of registration is an important step in the design of the dataset, because, for a fusion between images acquired at different times (or even different sensors), all of them must be perfectly matched (registered). The process of image registration used in this paper consists of taking both images (reference and the image to register) and finding several key points by using Oriented FAST and Rotated BRIEF (ORB) descriptors [18] which first use Features from Accelerated Segment Test (FAST) [19] to find key points, then apply Harris corner measure to find the top *K* points among them, and finally use a pyramid to produce multiscale-features. The number of points *K* is a parameter that must be selected carefully for an optimal registration. The Mean Square Error (MSE) is the selected metric we use to estimate the parameter *K*. Our proposal is to increase *K* as far as the MSE is not significantly decreased. The MSE results of the reference image (Aug 24th) concerning the other two images before and after being registered with respect to it (Sep 05th and Dec 22nd, the temporally farthest images considered) and actual not registered are shown in Table 2, with the best results in boldface.

**Table 2:** MSE measurement from the registration process with different values of *K.*

Date	Not Reg.	Reg *K* = 100	Reg *K* = 500	Reg *K* = 1000
Sep-05	2066.36	4530.89	1038.80	**1034.97**
Dec-22	2024.60	5002.19	**1724.10**	1811.11

**Fig. 4:**
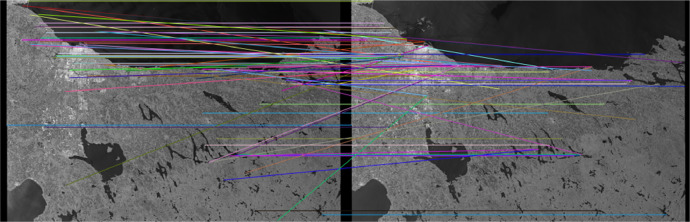
Matches of one of the registration processes.

As expected, the MSE of the registered images is much lower than that of the not-registered ones. The number of points chosen for this paper for the image registration is *K* = 500. The results of one of the registration operations and the corresponding matches are shown in Fig. 4.

#### 3.3.3 Multitemporal fusion

The fusion of the ten registered images is performed by averaging all of them, pixel by pixel, resulting in one single image. This resulting ‘super image’ will have the same dimensions and resolution as each one from the original SAR images. In publications like [20], an analysis of the effects produced by different approaches for building training datasets, based on different philosophies is carried out, including the synthetic, multitemporal and hybrid cases. In [21] generative model based on the decomposition of the Single Look Complex (SLC) images into a speckle component and a dominant scatteredcomponent was introduced, improving the despeckling performance achieved by mono-date networks. The networks were trained directly on SAR images, without ground truth, producing restored images of higher quality compared to the state-of-the-art techniques.

In order to measure the quality of the resulting image, the number of looks (ENL) is calculated on a 20 × 20 region of the image. This region has to be homogeneous, and the ENL is calculated according to Eq. (2).

(2) $$\text{ENL}=\frac{\mu^{2}}{\sigma^{2}}$$

The resulting ENL of the original SAR image was 16.490, while the resulting fusion (generated ground truth) was 70.605. This metric, the ENL, is the most used for SAR when no ground truth is available.

#### 3.3.4 Clip image

One last step for designing this ground truth dataset is clipping, which means that three parameters must be chosen to divide the actual SAR image (noisy) and the ground truth (not noisy or averaged) into several small images, so avoiding overlapped images (to get full use of the original SAR data). The clipping parameters are: the desired width of the clipped images (W), the desired height of the clipped images (H) and the stride from one clipped image to the next (S). A recommended setting of these parameters would be *W=H=S*= 512, which means that images of 512 × 512 pixels will be clipped, and the stride of 512 means that the next image will not have a pixel in common with the previous. With this setting, from the image shown in Fig. 1 which has 26,019 × 16,732 pixels, it is possible to obtain 1600 clipped images of 512 × 512 pixels. If a smaller stride is defined, the images will have the same size but significantly more images will be obtained, but it must be kept in mind that some pixels will be repeated between images in the same neighborhood, which should not be a problem and could be considered a data augmentation technique, a common practice in artificial intelligence and deep learning. The size of the image should be a power of 2, because of the dimension-reducing steps in DL, such as max pooling with a stride of 2, will downsample the image and divide its dimensions by 2.

**Fig. 5:**
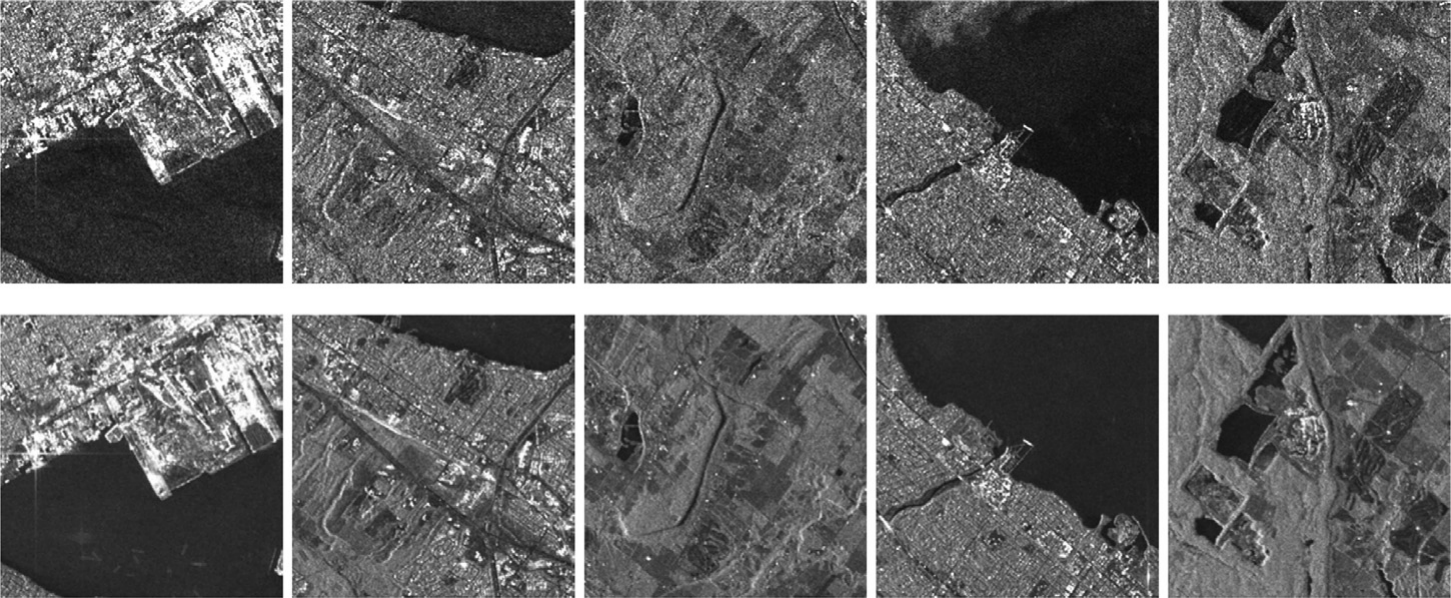
Example of five noisy images from the ‘*Noisy*’ subfolder (up) and their corresponding noiseless images from the ‘*GTruth*’ subfolder (down).

In general, in this proposed framework, an image of width W, height H, and stride S, can be clipped into a defined number of samples according to $N_{samples}=\left\lfloor W/S\right\rfloor \cdot \left\lfloor H/S\right\rfloor$, where ⌊x⌋ denotes the floor function, which maps x∈ℜ to the greatest integer less than or equal to x. Some examples of clipped noisy images with their corresponding ground truth after the multitemporal fusion process are shown in Fig. 5.

Some experiments and application performed by using the proposed methodology in this paper are described in [22] and [23]. The code used in the previous sections is available on https://github.com/rubenchov/SAR_despeckling_dataset, and the dataset with the structure described in Fig. 1 is available on https://data.mendeley.com/datasets/2xf5v5pwkr/1.

## Limitations

• The SAR images downloaded from Sentinel-1 have a resolution of 10 m per pixel, which, while adequate for many applications, may fall short of meeting the demands of other applications that require more detailed images. SAR images featuring superior technical specifications can be obtained from alternative sensors, but it must be noted that such resources typically entail associated costs and are not available for free download.
• The SAR images downloaded are 16,732 (height) by 26,019 (width). This substantial size poses a significant computational cost, both to handle and process. The images were clipped into smaller segments, each of them measuring 512 × 512 pixels. Consequently, to train deep learning models with this dataset, the dimensions of its input layer must align with this standardized size.
• During the clipping process, 1600 images were generated from the large image after the average process. The resulting images were split into a training set comprising 1500 images and a validation set comprising 100 images. This dataset size proves to be sufficiently large for training deep learning models, such as the autoencoder referenced in [22]. However, more complex models will surely require a larger dataset. There are two possible strategies to consider: adjusting the stride (S) to clip the large image into smaller ones, or downloading more images from an alternative geographic region employing the same sensor.
• In the process of designing this dataset, only Level 1 SAR images sourced from Sentinel-1 were utilized. Consequently, any model on this dataset will inherently incorporate knowledge from the speckle characteristics of these images.

## Ethics Statement

The authors confirm that, after reading the ethical requirements for publication in Data in Brief, the current work does not involve human subjects, animal experiments, or any data collected from social media platforms.

## CRediT authorship contribution statement

**Rubén Darío Vásquez-Salazar:** Conceptualization, Methodology, Writing – original draft. **Ahmed Alejandro Cardona-Mesa:** Methodology, Writing – original draft. **Luis Gómez:** Conceptualization, Methodology, Formal analysis, Writing – review & editing. **Carlos M. Travieso-González:** Conceptualization, Methodology, Formal analysis, Writing – review & editing. **Andrés F. Garavito-González:** Software, Investigation, Resources, Writing – original draft. **Esteban Vásquez-Cano:** Software, Investigation, Resources, Writing – original draft.

## Data Availability

SAR despeckling filters dataset (Original data) (Mendeley Data)
